# Supplementary material for: Controlled Release of Phycocyanin in Simulated Gastrointestinal Conditions Using Alginate-Agavins-Polysaccharide Beads
Source: Foods. 2023 Aug 31;12(17):3272. doi: 10.3390/foods12173272 (PMC10486973; doi:10.3390/foods12173272)

Table S1. Morphometric characterization of matrix-type encapsulates.

Parameter	Formulated matrix for ion gelation encapsulation							
	M1		M2		M3		M4	
	Control	CPC	Control	CPC	Control	CPC	Control	CPC
Area (mm^2)	0.172 ± 0.004	0.204 ± 0.022	0.410 ± 0.035	0.466 ± 0.041	0.966 ± 0.075	1.152 ± 0.006	1.150 ± 0.036	1.180 ± 0.030
Perimeter (mm)	1.584 ± 0.034	1.722 ± 0.081	2.758 ± 0.167	2.684 ± 0.195	3.926 ± 0.194	4.384 ± 0.240	4.088 ± 0.054	4.166 ± 0.049
Feret Diameter (mm)	0.500 ± 0.008	0.552 ± 0.028	0.896 ± 0.066	0.866 ± 0.063	1.328 ± 0.078	1.400 ± 0.079	1.352 ± 0.027	1.180 ± 0.030
Circularity	0.854 ± 0.016	0.858 ± 0.009	0.690 ± 0.050	0.824 ± 0.054	0.788 ± 0.029	0.774 ± 0.072	0.864 ± 0.007	0.854 ± 0.006
Solidity	0.988 ± 0.002	0.990 ± 0.000	0.976 ± 0.009	0.980 ± 0.013	0.992 ± 0.002	0.982 ± 0.016	0.998 ± 0.002	1.000 ± 0.000

The values represent the mean ± SEM of photomicrographs processed with the open-source software ImageJ. Control: encapsulates formulated without C-phycocyanin (CPC). CPC: encapsulates formulated with 20 mg/mL of CPC. M1: alginate; M2: alginate and agavins; M3: alginate, agavins, and κ-carrageenan; M4: alginate, agavins, κ-carrageenan, and carboxymethyl cellulose.

Table S2. Release mechanisms of C-phycocyanin from different encapsulates.

		Zero order		First order		Korsmeyer–Peppas		
		k	R^2	k	R^2	n	kp	R^2
M1	SGP	0.0058	0.8320	0.0205	0.948	0.2248	0.2156	0.9756
	SIP	0.0048	0.9421	0.0089	0.8705	0.0398	0.7945	0.5364
M2	SGP	0.0070	0.7991	0.0122	0.8695	0.1551	0.3508	0.9980
	SIP	0.0047	0.9462	0.0144	0.9764	0.0699	0.6524	0.9793
M3	SGP	0.0073	0.8339	0.0134	0.8634	0.2586	0.2327	0.9494
	SIP	0.0046	0.9587	0.0136	0.9863	0.1973	0.3266	0.8402
M4	SGP	0.0071	0.8973	0.0128	0.9505	0.3381	0.1558	0.9925
	SIP	0.0048	0.9502	0.0181	0.9755	0.1117	0.5357	0.7777

M1: alginate; M2: alginate and agavins; M3: alginate, agavins, and κ-carrageenan; M4: alginate, agavins, κ-carrageenan, and carboxymethyl cellulose.

Figure S1. Brightfield photomicrographs of the encapsulates obtained with 20 mg/mL CFC. M1: alginate; M2: alginate and agavins; M3: alginate, agavins and κ-carrageenan; M4: alginate, agavins, κ-carrageenan and carboxymethylcellulose. CFC: C-phycocyanin.

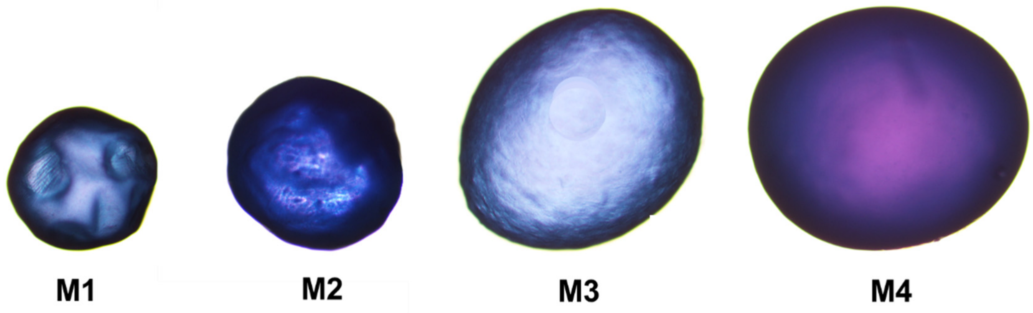

Figure S2. Correlation between the released CPC concentration during the digestive process (Right Y-axis) and the brightness of the bead's surfaces (Left Y-axis), over time. Each point represents the mean of 5 measurements ± SEM. Arrows indicate the points where brightness values and released CPC intersect for each formulated encapsulating matrix (M1, M2, M3, and M4).

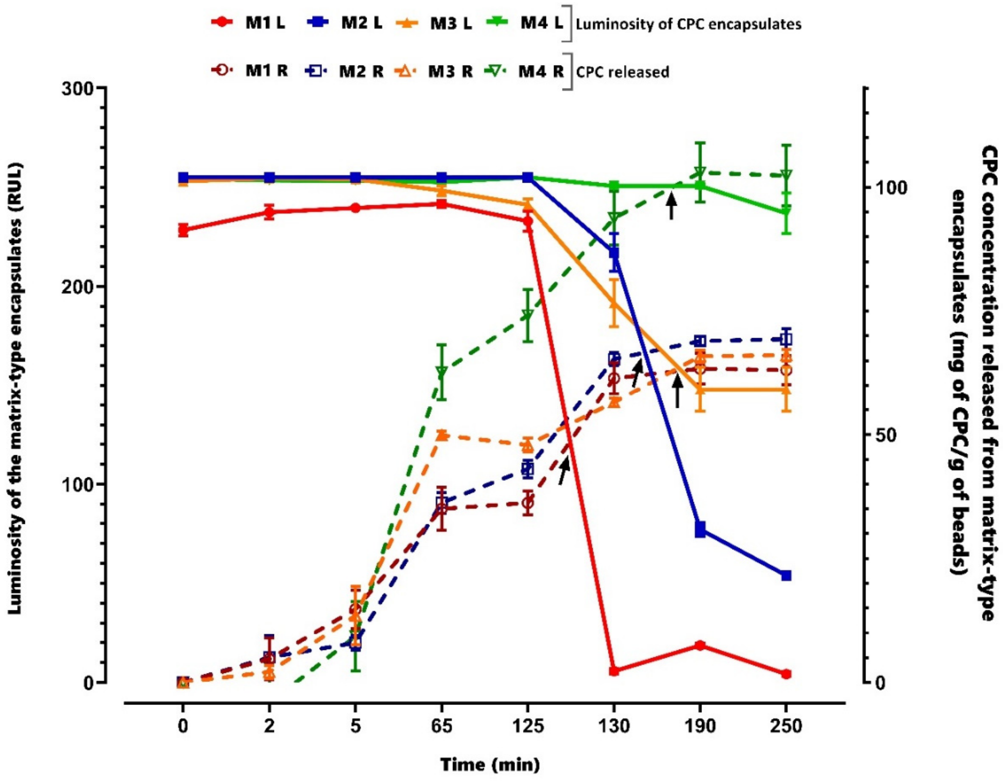

Supplement: Supplementary file 1 [file foods-12-03272-s001.zip › foods-2535945-supplementary.pdf]
